# Eco-friendly extraction of collagen from beluga sturgeon skin using ultrasound-assisted deep eutectic solvents: efficiency and characterization

**DOI:** 10.1016/j.fochx.2025.102988

**Published:** 2025-09-03

**Authors:** Elahe Taghitabar Malekshah, Masoud Rezaei, Shahab Naghdi, Reza Tahergorabi

**Affiliations:** a Seafood Processing Department, Marine Sciences Faculty, Tarbiat Modares University, Noor, Iran; b Food and Nutritional Sciences Program, North Carolina Agricultural and Technical State University, Greensboro, NC, USA

**Keywords:** Collagen extraction, Deep eutectic solvents, Green technology, Beluga skin, Ultrasound treatment, Structural and functional properties

## Abstract

The current study aims to extract collagen from the skin of beluga sturgeon (*Huso huso*) using deep eutectic solvents combined with ultrasound as a green recovery method. It evaluates both the yield of the extraction process and the structural and physicochemical properties of the extracted collagen. The extraction utilized deep eutectic solvents, including choline chloride-glycerol, choline chloride-urea, and choline chloride-acetic acid, all at a molar ratio of 1:2. Among these, the choline chloride-acetic acid solvent yielded the highest extraction rate at 20.28 % and was selected for further ultrasound treatments lasting 5, 10, 15, and 20 min, with a power setting of 400 W. Ultrasound treatment significantly enhanced collagen extraction yield, with a 10-min treatment achieving the highest efficiency at 24 %. SDS-PAGE analysis confirmed the presence of type I collagen, indicating no structural changes during extraction. FTIR analysis showed that the triple helical structure of ultrasound-treated collagen was preserved throughout the process. Longer ultrasound treatment durations improved collagen purification, resulting in higher protein and total amino acid content. Additionally, ultrasound application enhanced thermal stability, water holding capacity, and gel formation power, with peak values observed during a 15-min treatment. In summary, extracting collagen from beluga skin using deep eutectic solvents in combination with ultrasound is an effective method for enhancing both yield and functionality of the resulting collagen.

## Highlights

• Ultrasound-assisted deep eutectic solvents boosted collagen yield from beluga skin.
• Choline chloride-acetic acid was the most effective green solvent for extraction.
• Collagen retained its native triple-helix structure and type I integrity after treatment.
• Ultrasound improved collagen's thermal stability, water holding, and gel strength.
• The method offers a sustainable, efficient alternative to conventional collagen extraction.

## 1 Introduction

The growing global population's need for protein has fueled a massive expansion in aquaculture and seafood processing. The beluga sturgeon (*Huso huso*) is one of the most significant fish species in aquaculture, particularly along the southern Caspian Sea coasts. Valued for their meat and caviar, which command high prices in the global market, belugas also provide valuable by-products such as skin, cartilage, and fins (Maryam Atef et al., 2020). These by-products can be utilized to produce collagen-based biomaterials, enhancing the economic viability of sturgeon aquaculture while simultaneously reducing environmental pollution associated with waste disposal (Xi Zhang et al., 2014). The increasing demand for caviar and meat has driven a rise in sturgeon farming, with projections indicating substantial growth in the beluga caviar market due to consumer preferences for sustainable food sources (Maryam Atef et al., 2020).

Collagen is among the most abundant compounds present in fish byproducts, accounting for approximately 30 % of the total protein content in vertebrates (Bisht et al., 2021). Even though researchers have discovered and categorized more than 28 types of collagen, it is evident that each type possesses distinct characteristics, with type I collagen being the most abundant. (Pezeshk et al., 2022). Collagen has numerous applications in the food, cosmetic, and biomedical industries due to its exceptional qualities, including its gelling ability, anti-ageing effects, bioactivity, and role in wound healing (Silva et al., 2024). As demand for collagen grows, the extraction process is increasingly on the rise, and it is projected that the global collagen market will reach $6.63 billion by 2025(Ahmed et al., 2020). Collagen is commercially obtained from the skin of cows and pigs; however, these sources may carry health risks and face religious limitations. As a result, marine-derived collagen has gained attention as a safer and more universally acceptable alternative (Huang et al., 2016). Conventional extraction methods, such as acid or enzymatic hydrolysis, are often inefficient, time-consuming, and may result in structural degradation of collagen, leading to poor yield and quality (Moreira-silva et al., 2016). These challenges have prompted the search for greener and more effective alternatives. Deep Eutectic Solvents (DES) are an emerging class of environmentally friendly solvents formed by mixing a hydrogen bond acceptor (HBA) and a hydrogen bond donor (HBD), resulting in a eutectic mixture with a melting point lower than that of the individual components. DES possess several desirable properties including biodegradability, low toxicity, low volatility, tunable polarity, and cost-effectiveness (Hansen et al., 2021). These features make DES promising candidates to replace harsh chemical solvents in biomolecule extraction processes. In collagen extraction, DES can potentially enhance performance by disrupting collagen-fiber matrices, solubilizing proteins under mild conditions, and preserving molecular integrity. Moreover, different DES compositions—depending on the type of HBA and HBD—can exhibit varying degrees of polarity, viscosity, and hydrogen bonding capacity, which in turn affect their extraction efficiency (Bisht et al., 2021). In addition, ultrasound-assisted extraction was employed to further enhance collagen recovery. Ultrasound generates cavitation forces that disrupt tissue matrices and facilitate solvent penetration, often leading to higher yield and shorter processing times, while preserving protein structure (Lu et al., 2023). This study aimed to investigate the application of different deep eutectic solvent (DES) systems, both individually and in combination with ultrasound-assisted extraction, for the sustainable and efficient extraction of collagen from beluga sturgeon skin, with a focus on evaluating their effects on yield, physicochemical properties, structural integrity, and functional characteristics of the extracted collagen. The importance of this research lies in addressing the critical need for eco-friendly and scalable alternatives to conventional extraction methods, which often involve hazardous chemicals and high energy consumption. By developing a greener and more efficient approach, this study contributes to advancing sustainable marine resource utilization, offering significant potential for applications in biomedicine, cosmetics, and food industries while aligning with global demands for environmentally responsible production processes.

## 2 Materials and methods

### 2.1 Preparation of samples

Beluga skin was provided by the Babolsar Fisheries Department's sturgeon processing center. The fish skin was completely covered with ice and transferred to the laboratory of Tarbiat Modares University. The fish skin was cleaned by removing the meat, rinsed with cold distilled water, and kept frozen at −18 °C. The skin samples were cut into smaller pieces and freeze-dried for 48 h. Afterward, the dried samples were ground into powder using an industrial grinding machine. The powdered sample was stored at −18 °C.

### 2.2 Composition analysis of the beluga skin

To measure the moisture content, the skin sample was placed in a freeze-dryer for 48 h. The percentage of moisture was then calculated based on the weight difference. For ash content measurement, 1 g of freeze-dried skin powder was placed in an electric oven at a temperature of 600 °C for 4 h. Afterward, the resulting ash was weighed, and the percentage of ash was calculated. The fat content was measured using a Soxhlet apparatus, while the protein content was determined using the Kjeldahl method.

### 2.3 Preparation of eutectic solvents

The eutectic solvents used in the present study were prepared based on the information provided in Table 1. The first solvent, consisting of choline chloride and glycerol in a molar ratio of 1:2, was produced by adding 20 % by weight of distilled water (Gao et al., 2018). The ingredients were stirred in an oil bath at 60 °C for 2 h (Hong Zhu, n.d.). The second solvent, composed of choline chloride and urea in a molar ratio of 1:2, was prepared by stirring the components in a water bath at 60 °C for 1 h, also with the addition of 20 % by weight of distilled water. The third solvent, containing choline chloride and acetic acid in a molar ratio of 1:2, was made by adding 20 % by weight of distilled water and stirring in a water bath at 70 °C for 2 h and 30 min (Virginia et al., 2021) (Table 1).

**Table 1:** Molar ratio of deep eutectic solvents.

Hydrogen donor	Hydrogen acceptor	Hydrogen acceptor/hydrogen donor
Glycerol	Choline chloride	1:2
urea	Choline chloride	1:2
Acetic acid	Choline chloride	1:2

### 2.4 Collagen recovery using eutectic solvents

Freeze-dried skin powder was mixed by DES (1,10 *w*/w) by stirring in a 45 °C water bath for 1 h, followed by centrifugation at 4665 ×*g* for 45 min. After centrifugation, two phases are obtained: a liquid phase and a solid phase. The supernatant is separated and placed in dialysis bags against distilled water for 72 h, with the distilled water changed every 12 h. The obtained extract is freeze-dried for 48 h, after which the material is ground into powder using an electric grinder. Then, the treatment with the highest extraction yield was used for further evaluation in combination with ultrasound treatment.

### 2.5 Applying ultrasound to the selected solvent based on the yield of recovered collagen

The treatment with the highest efficiency, using choline chloride-acetic acid, was subjected to ultrasound treatments with durations of 5, 10, 15, and 20 min, at a power of 400 W with a constant temperature of 20 °C, operating in an on-off cycle of 7 s on and 3 s off. To do this, the dried skin powder was then mixed with the selected eutectic solvent. This mixture was placed in a container covered by crushed ice to control the temperature during ultrasound treatment. After applying ultrasound, collagen was recovered according to the previously mentioned method.

### 2.6 Collagen recovery efficiency percentage and total protein content of the extract

Extraction efficiency based on dry weight was calculated using Eq. 1.

(1) Yield = weight of dry extract/weight of dry skins × 100

### 2.7 Evaluation of structural properties of collagen

#### 2.7.1 SDS-PAGE analysis of the extracted samples

Collagen samples, previously lyophilized and dissolved in 0.5 M acetic acid (2 mg/ml), were combined 1:1 (v:v) with Laemmli buffer (5 % β-mercaptoethanol). Denaturation was carried out at 70 °C for 10 min. Electrophoresis was conducted on a 7.5 % Mini-PROTEAN® TGX™ gel (12 wells, 20 μl) at 200 *V* for 30 min using 10 μl of each sample and a protein ladder. Post-run, the gel was Coomassie-stained (1 h, stirred), washed overnight in distilled water, and imaged with a 48 MP RGB camera(Batista et al., 2022).

#### 2.7.2 Ultraviolet spectroscopy (UV–visible)

Following Pezeshk et al. (2022) (Pezeshk et al., 2022), the UV absorption of collagen was assessed by dissolving frozen samples in 0.5 M acetic acid and scanning from 200 to 400 nm using a UV-1800 spectrophotometer (Mapada Instruments, Shanghai, China).

#### 2.7.3 Fourier transform infrared spectroscopy (FTIR)

Beluga skin collagen was characterized by FT-IR spectroscopy (Vate et al., 2022) (Vate et al., 2022) using a Nicolet 6700 spectrometer. Lyophilized samples were scanned once (1400–4000 cm^−1^, 1 cm^−1^ resolution) at 25 °C on a crystal cell.

#### 2.7.4 Amino acid composition

The amino acid composition was determined by hydrolyzing collagen samples in 6 N HCl (110 °C, 24 h, *v*acuum). Hydrolyzed collagen were solved in 0.2 M citric acid. Following filtration, analysis was performed on a Hitachi L8900 amino acid analyzer (Japan) using established procedures (Tamilmozhi et al., 2013).

#### 2.7.5 Viscosity of collagen solution

The solution of 0.03 % *w*/*v* in 0.1 M acetic acid collagen underwent viscosity analysis with a Brookfield viscometer. 8 ml aliquot was measured while maintaining a constant spindle speed of 100 rpm. The solutions were heated at a rate of 20 to 60 °C, gradually increasing the temperature and maintaining it at each selected level for 30 min. The relative viscosity at each temperature was determined by comparing it to the viscosity obtained at 20 degrees Celsius, according to the following formula. Td is the temperature when viscosity drops to 50 % of its initial value during heating (Pati et al., 2010).

Fractional viscosity = (measured viscosity – minimum viscosity) / (maximum viscosity - minimum viscosity)

#### 2.7.6 Differential scanning calorimetry (DSC)

Thermal analysis was conducted using a PerkinElmer STA 6000 differential scanning calorimeter (P/N N5370217). Following a 5-min degassing period at 25 °C, collagen samples were subjected to a controlled heating ramp (10 °C/min) between 20 and 100 °C. The melting temperature (Tm) corresponded to the endothermic peak maximum in DSC thermograms, with denaturation parameters deri*v*ed through NanoAnalyze software processing.

### 2.8 Evaluation of the functional properties of collagen

#### 2.8.1 Gel strength measurement

Following British Standard BS 757 [15], collagen gel strength was assessed by hydrating 7.5 g lyophilized collagen in 105 ml water (6.67 % *w*/*v*). The solution was stirred for 1 h, incubated at 60 °C (30 min), then gelled at 4 °C (12–16 h). Texture analysis was performed using a TVT 300 XP instrument (TexVol) with P-Sp5 probe (5 g trigger), measuring peak penetration force (g) at 4 mm depth.

#### 2.8.2 Water holding capacity

WHC determination was performed according to reference (Akram & Zhang, 2020). Collagen samples (0.05 g) were combined with 5 ml distilled water in tared culture dishes (8 cm diameter) and maintained at 37 °C with 50 % relative humidity. Mass measurements were taken every 10 min over 70 min. The WHC percentage was derived from eq. (2):

(2) WHC (%) = (ΔW/W0) × 100

where ΔW as water mass variation and W0 as starting water quantity.

### 2.9 Statistical analysis

Statistical analysis was carried out using SPSS Statistics 20.0 for Windows. One-way ANOVA, followed by Duncan's test (*p* *<* *0.05*), was employed to compare the means of treatments, with triplicate data expressed as mean ± standard.

## 3 Results and discussion

### 3.1 Approximate composition analysis of beluga skin

The protein, fat and ash content of the beluga fish skin based on dry weight was 73.66 ± 0.14, 29.90 ± 0.68, and 1.55 ± 0.1, respectively (Table 2). This content reported in this study was almost similar to that of beluga skin in the study by Barimani et al. (2021) (Barimani et al., 2021).

**Table 2:** Approximate composition analysis of beluga skin (*Huso huso*).

Protein	Fat	Ash
73.66 ± 0.14	29.90 ± 0.68	1.55 ± 0.11

The data were expressed as mean value ± SD (*n* = 3) and reported based on dry weight.

### 3.2 Collagen recovery yield

The percentage of collagen recovery using each of the eutectic solvents is reported in the Table 3. As can be seen from the results, there was no significant difference between eutectic solvent treatments in terms of efficiency (*P* > 0.05). Among the eutectic solvents, choline chloride-acetic acid solvent has the highest efficiency (21.18 ± 3.93) numerically due to the acidity of the extraction medium and choline chloride urea solvent due to its high pH and distance from the acidic medium required for collagen extraction has achieved the lowest efficiency (18.61 ± 1.15) numerically in collagen extraction. These results were compared by Bisht et al. research which choline chloride urea had the lowest yield of collagen extraction because of its high PH (Bisht et al., 2021). Choline chloride-acetic acid solvent was considered as the chosen solvent for applying ultrasound due to having the highest numerical yield.

**Table 3:** Extraction yield percentage using different treatments of eutectic solvents.

Choline Chloride-Glycerol	Choline chloride-urea	Choline chloride-acetic acid
18.70 ± 1.00 ^a^	18.61 ± 1.15 ^a^	21.18 ± 3.93 ^a^

The results were expressed as mean value ± SD (*n* = 3). Different letters within the samples mean statistical difference (*p* < 0.05).

According to Table 4, treatment ChClUS10 had the highest extraction yield (25.66 ± 2.08), while treatment ChClUS20 had the lowest yield (19.82 ± 0.30). With increasing ultrasound time, collagen extraction yield increased numerically compared to the control. This increase may be attributed the phenomenon of cavitation during ultrasound application, which causes cell disruption, and mechanical energy is created that enhances acid penetration into the skin matrix to extract collagen (Pezeshk et al., 2022). The collagen yield decreased markedly with excessive ultrasonication time, as the associated mechanical shear and localized heating promoted molecular degradation. These findings corroborate the work of Hu et al.(Hu et al., 2023).

**Table 4:** Extraction yield percentage using choline chloride and different ultrasound treatments.

Ch Cl- AA- 10 min US	Ch Cl- AA- 15 min US	Ch Cl- AA- 20 min US
25.66 ± 2.08 ^b^	22.35 ± 1.62 ^a^	19.82 ± 0.30 ^a^

The results were expressed as mean value ± SD (n = 3). Different letters within the samples mean statistical difference (p < 0.05).

### 3.3 Molecular weight analysis of the extracted collagens by SDS-PAGE

**Fig. 1:**
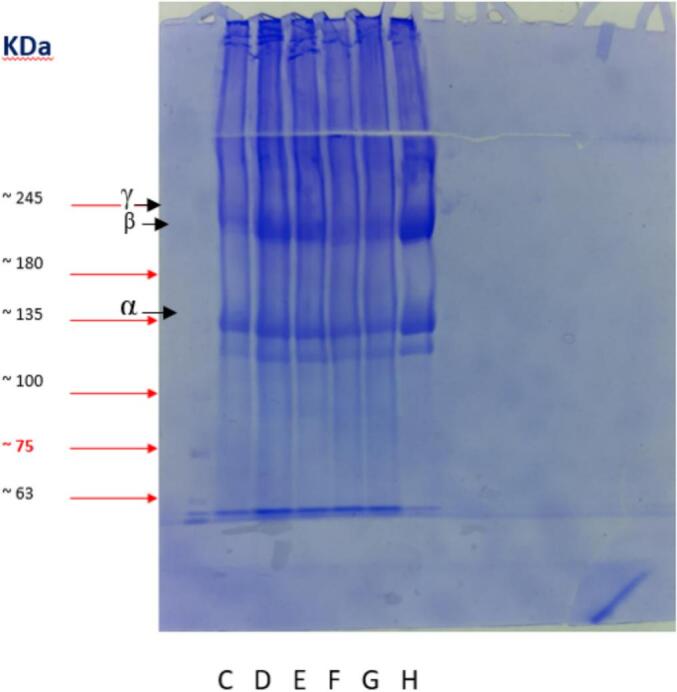
Protein pattern of collagen extracted from beluga skin (C: Choline Chloride- Acetic acid, D: Choline Chloride- Acetic acid 5 min ultrasound, E: Choline Chloride- Acetic acid- 10 min ultrasound, F: Choline Chloride- Acetic acid- 15 min ultrasound, Choline Chloride- Acetic acid- 20 min ultrasound).

The molecular weight of the α, β and γ bands were 180 kDa, 220 kDa and 240 kDa, respectively (See Fig. 1), which aligns with the study by Heidari and Rezaei (2022). (Heidari & Rezaei, 2022). Based on the combination of β and γ and the molecular weight of the collagens, which are similar to type I collagen extracted from salmon skin, the extracted collagens can be classified as type I. Additionally, the ratio of α1 to α2 chains in all samples was about 2:1, indicating type I collagen (Vate et al., 2022). The molecular weight distribution of collagen bands affects their quality. No significant difference was seen in bands between different treatments. These results demonstrate that all collagen treatments are pure and that the integrity of their structure is maintained during ultrasound application (Pezeshk et al., 2022). No low molecular weight protein was detected in the samples, suggesting that ultrasound does not negatively affect the polypeptide structure of the extracted collagens (Vate et al., 2022). With longer ultrasound exposure (treatments F and G), the β band appeared fainter. Intense ultrasound energy applied for extended duration may convert the β band into an α chain by weakening the intermolecular and intramolecular crosslinks in the telopeptide region, which corroborates the findings of this observation (Heidari & Rezaei, 2022).

### 3.4 UV–vis spectrophotometry

**Fig. 2:**
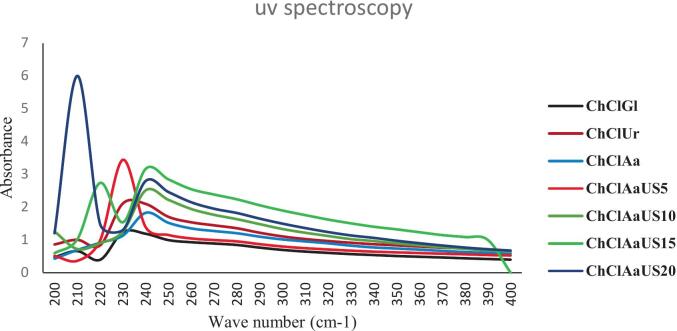
UV spectroscopic diagram of collagen extracted from beluga skin (ChClGl: Choline Chloride- Glycerol, ChClUr: Choline Chloride- Urea, ChClAa: Choline Chloride- Acetic acid, ChClAaUS5: Choline Chloride- Acetic acid- 5 min ultrasound, ChClAaUS10: Choline Chloride- Acetic acid- 10-min ultrasound, ChClAaUS15: Choline Chloride Acetic acid 15-min ultrasound, ChClAaUS20: Choline Chloride 20-min ultrasound).

Fig. 2 illustrates the UV absorption spectra of the extracted collagen samples. All treatments exhibited a prominent absorption peak centered around 235 nm, which is characteristic of collagen and corresponds to the absorption of the triple-helical structure and peptide bonds within the polypeptide chains. This absorption peak typically appears between 220 and 280 nm in collagen samples and is mainly attributed to the presence of functional groups such as COOH, CONH₂, and C=O in the collagen molecules (Chen et al., 2016; Heidari & Rezaei, 2022). The distinct peak near 235 nm confirms the preservation of the collagen's triple helix structure, which is crucial for its functional properties. Additionally, there is absorption observed in the 200–220 nm range, which can be attributed to the peptide bonds in the collagen backbone, further validating the proteinaceous nature of the samples. Importantly, the spectra show minimal absorbance at 280 nm, a wavelength commonly associated with aromatic amino acids such as tyrosine and tryptophan. The low absorption at 280 nm indicates a low content of these amino acids in the collagen samples, consistent with the known amino acid composition of collagen, which is typically poor in aromatic residues. This feature also suggests that the extracted collagen is of high purity with minimal contamination from other proteins rich in aromatic amino acids. These UV absorption characteristics align well with previous studies on collagen extraction and characterization (Chen et al., 2016; Heidari & Rezaei, 2022; Liu et al., 2023), confirming the successful isolation of collagen with intact triple-helical structure and high purity. Thus, the UV spectra provide strong evidence that the extraction process preserved the structural integrity of collagen while minimizing impurities.

### 3.5 Infrared spectroscopy or Fourier transform (FTIR)

**Fig. 3:**
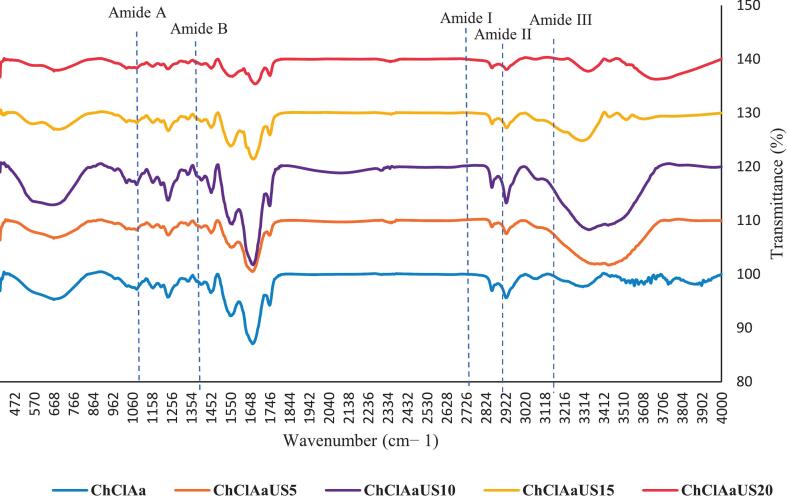
FTIR diagram of collagen extracted from beluga skin (ChClGl: Choline chloride- glycerol, ChClUr: Choline Chloride- urea, ChClAa: Choline Chloride- Acetic acid, ChClAa US 5: Choline Chloride Acetic acid- 5 min ultrasound, ChClAaUS10: Choline Chloride Acetic acid- 5 min ultrasound, ChClAaUS15: Choline Chloride Acetic acid- 15 min ultrasound, ChClAaUS20: Choline Chloride Acetic acid- 20 min ultrasound).

The FT-IR spectra of the samples, recorded in the range of 400 to 4000 cm^−1^, are illustrated in Fig. 3. The vibrations in amide bands I (1700–1600 cm^−1^), II (1550–1600 cm^−1^), and III (1300–1200 cm^−1^) are typical for collagen and related to the C—O stretching vibration. A lower wavenumber usually suggests the formation of a hydrogen bond involving the N—H stretch, with the CO residue playing a crucial role in stabilizing the triple helix structure. The N—H bend is indicated by the amide II band. When the amide I and II bands of collagen shift to lower wavenumbers, it signifies the formation of additional hydrogen bonds within the triple helix structure. Collagen's intricate intermolecular interactions involve Amide III, which is a mixture of C—N stretching and N—H deformation. The N—H stretching vibration is contributes to the absorption band of amide A, while the CH_2_ asymmetric stretching is responsible for the band of amide B. The absorption peak of amide A is located between 3400 and 3440 cm^−1^, although it may shift slightly to lower wavenumbers as the protein's NH group forms intermolecular hydrogen bonds. Increased hydrogen bonding engagement with collagen peptides results in shorter wavelengths, suggesting enhanced structural consistency of the triple helix. Collagen's triple helical stability was assessed by calculating the amide III-to-1450 cm^−1^ band intensity ratio from FTIR spectra, where a ratio of ∼1.0 served as the stability criterion (Vate et al., 2022).

The amide I band was detected at wavelengths of 1661, 1662, 1663, 1659, 1662, 1671, and 1662 cm^−1^ for treatments ChClAaUS0, ChClAaUS5, ChClAaUS10, ChClAaUS15, ChClAaUS20, and AaUS0, respectively. The amide II band was observed at wavelengths of 1553, 1558, 1559, 1548, 1560, and 1549 cm^−1^ for the same treatments. The amide III band appeared at wavelengths of 1237, 1240, 1238, 1240, and 1237 cm^−1^, respectively, for treatments ChClAaUS0, ChClAaUS5, ChClAaUS10, ChClAaUS15, ChClAaUS20, and AaUS0. The amide A band was detected at wavelengths of 3565, 3436, 3400, 3304, 3335, and 3309 cm^−1^ for treatments ChClAaUS0, ChClAaUS5, ChClAaUS10, ChClAaUS15, ChClAaUS20, and AaUS0. Lastly, the amide B band was observed at wavelengths of 2962, 2926, 2926, 2927, 2927, and 2924 cm^−1^ for the respective treatments. In the FTIR spectrum of beluga skin collagen, five main absorption peaks related to amide A, I, II, and III were identified, similar to the collagen structure extracted in other studies (Liu et al., 2023). FTIR analysis confirms that the triple helical structure of ultrasound-treated collagen remains intact throughout extraction. This preservation is attributed to pyrrolidine rings' ability to stabilize the polypeptide chain's secondary structure(Pezeshk et al., 2022).

### 3.6 Amino acid profile

The amino acids glycine, proline, hydroxy proline, and alanine are typically abundant in collagen. Glycine, the main amino acid in type I collagen, is crucial for maintaining the helical structure. Glycine appears as every third amino acid residue in collagen's distinct amino acid sequence, which explains this. Collagen subunits' amino acid sequence is made up of Glycine-X-Y repetitions, in which Y is variable but usually contains hydroxyproline, and X is variable but usually contains proline (Vate et al., 2022). The highest concentrations of amino acids observed for alanine, arginine, glycine, proline, and hydroxyproline respectively. All these amino acids increased proportionally with longer sonication times. All samples exhibited the lowest levels of histidine and isoleucine, and these results were comparable to those of other studies (Heidari & Rezaei, 2022). Collagen has two distinctive amino acids; proline and hydroxyproline, which are crucial for preserving its structural integrity. Collagens with higher proline and hydroxyproline contents have higher mechanical strengths, improved thermal stability, and higher thermal denaturation temperatures. By creating pyrrole rings and hydrogen bonding, this improvement fortifies the helical structure (Tamilmozhi et al., 2013). Prolonged ultrasound exposure improves collagen purification through enhanced molecular disaggregation, ultimately increasing the extractable protein fraction and amino acid liberation.

However, the application of ultrasound led to a decrease in the amino acid content (specifically proline and hydroxyproline) in collagens, which aligns with the findings of Vate et al. (Vate et al., 2022). A reduction in the content of imino acids results in decreased thermal stability of collagen. Therefore, treatments without ultrasound yielded the highest levels of imino acids, indicating greater thermal stability. This revision enhances clarity and flow while ensuring that the scientific content remains intact.

### 3.7 Viscosity of collagen solution

**Fig. 4:**
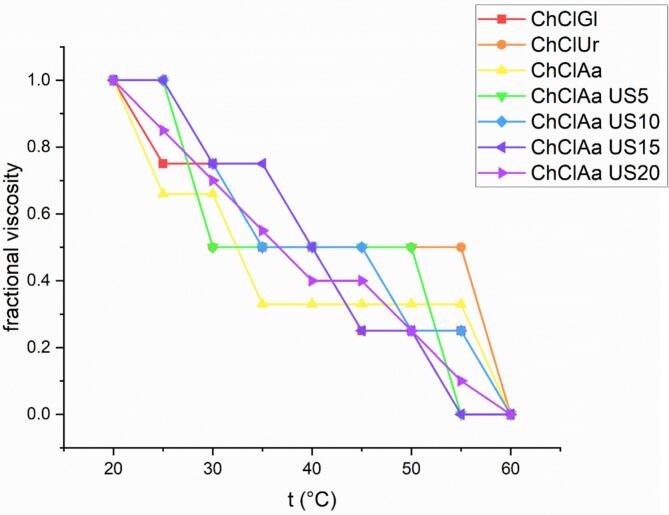
Viscosity diagram of collagen extracted from beluga skin (ChClGl: Choline chloride- glycerol, ChClUr: Choline Chloride urea, ChClAa: Choline Chloride Acetic acid, ChClAaUS 5: Choline Chloride Acetic acid- 5 min ultrasound, ChClAa US10: Choline Chloride Acetic acid- 5 min ultrasound, ChClAaUS15: Choline Chloride Acetic acid- 15 min ultrasound, ChClAa US20: Choline Chloride Acetic acid- 20 min ultrasound).

Fig. 4 displays the temperature-dependent fractional viscosity profile of collagen samples across the 20–60 °C range. The results indicate that the fractional viscosity of the samples decreases as the temperature increases, consistent with findings from Pezeshk et al. (2022) (Pezeshk et al., 2022). The denaturation temperature (Td) represents the thermal threshold at which collagen's organized triple-helical conformation undergoes irreversible unfolding into disordered random coil configurations. At this denaturation temperature, the fractional viscosity is expected to be 0.5 (Pati et al., 2010). The denaturation temperature (Td) in solution and the shrinkage temperature (Ts) of the fiber are typically are often employed to assess the thermal stability of collagen. The temperature at which the collagen triple helix structure in solution changes to random coils is known as the Td (Yan et al., 2008). According to the research, collagen from beluga fish skin has a triple helix structure that is less thermally stable than collagen from land mammals. This difference is primarily attributed to variations in body temperature and habitat temperature between these species. Furthermore, a reduction in imino acid content correlates with a decrease in denaturation temperature (Pezeshk et al., 2022).

### 3.8 Differential scanning calorimetry (DSC)

**Fig. 5:**
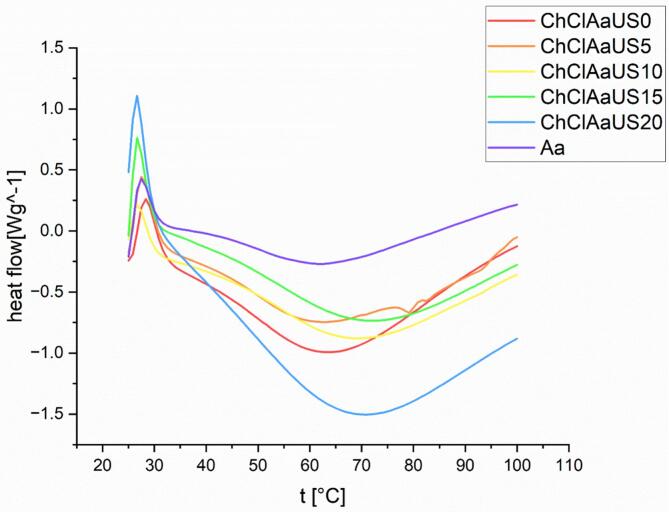
Differential calorimetry (DSC) diagram of collagen extracted from beluga fish skin from beluga skin (ChClGl: Choline chloride- glycerol, ChClUr: Choline Chloride urea, ChClAa: Choline Chloride Acetic acid, ChClAaUS 5: Choline Chloride Acetic acid- 5 min ultrasound, ChClAa US10: Choline Chloride Acetic acid- 5 min ultrasound, ChClAaUS15: Choline Chloride Acetic acid- 15 min ultrasound, ChClAa US20: Choline Chloride Acetic acid- 20 min ultrasound).

DSC thermograms of recovered collagen are represented in Fig. 5. DSC thermograms of collagen-II exhibited two distinct endothermic transitions corresponding to (1) triple helix denaturation (Td) and (2) complete molecular unfolding (Tm) during thermal ramping (Akram & Zhang, 2020). Tmax represents the critical shrinkage temperature where collagen fibers contract to 33 % of their original length, accompanied by a phase transition from solid to liquid state. According to Table 5, After applying ultrasound for five minutes in ChChlAaUS5 treatment, the Tmax of extracted collagen decreased, most likely as a result of the cavitation and turbulence of ultrasound probe intense. Covalent crosslinks of Collagen molecules can be broken by these energies. Prolonged ultrasound treatment for 10, 15, and 20 min in treatments ChChAaUS10, ChClAaUS5, and ChClAaUS15 increased the thermal stability of the collagen samples, suggesting that extended ultrasound exposure may enhance the aggregation of collagen particles by forming various types of covalent bonds. The highest Tmax was observed in treatment ChChAaUS10 (10 min). These results align with findings from Pezeshk et al. (2022). As the duration of ultrasound application increased, Td decreased. Treatment ChChAaUS0 (no ultrasound) exhibited the highest Td, while treatment ChClAaUS20 (20 min of ultrasound) had the lowest. The amounts of proline and hydroxyproline supported this declining trend in Td, which was linked to the amino acid profile. The structure, triple helix interchain hydrogen bonding, and thermal denaturation of the protein were all affected by amino acid concentration (Minh Thuy et al., 2014). The heat resistance of collagen is greatly impacted by its proline and hydroxyproline content, as these amino acids help form stabilizing cross-links between collagen molecules. Variations in thermal stability among collagens from different species are attributed to differences in proline and hydroxyproline content, as well as ambient and body temperatures. The pyrrolidine rings in proline and hydroxyproline residues enhance collagen's thermal stability, with hydroxyproline's hydroxyl group providing additional stabilization through hydrogen bond formation (Kaewdang et al., 2014). Compared to various aquatic animal collagens, terrestrial animal collagen extracted via ultrasound demonstrated higher thermal denaturation temperatures (Td). Body temperature and environmental conditions significantly impact thermal stability (Akram & Zhang, 2020).Interestingly, prolonged ultrasound treatment (10–20 min) led to an increase in Tmax (shrinkage temperature), likely due to enhanced fiber aggregation and physical cross-linking induced by mechanical forces. In contrast, the denaturation temperature (Td) decreased, which may be attributed to a reduction in imino acid content (proline and hydroxyproline), critical for stabilizing the triple-helical structure of collagen. This apparent contradiction reflects the dual effect of ultrasound: while it weakens hydrogen-bonded helix stability (decreasing Td), it simultaneously promotes non-covalent aggregation (increasing Tmax). Such opposing thermal behaviors are consistent with the multifaceted structural response of collagen to sonication and have been reported in prior studies.

**Table 5:** Amino acid composition (residues per 1000 residues) of collagen extracted from beluga skin using different DES-ultrasound treatments.

Amino Acid	Ch Cl – AA	Ch Cl – AA 5 min US	Ch Cl- AA 10 min US	Ch Cl- AA 15 min US	Ch Cl- AA 20 min
Aspartic Acid	70.13	61.73	67.31	63.26	64.6
Glutamic Acid	104.14	99.31	105.45	103.36	102.62
Asparagine	0.0	0.0	0.0	0.0	0.0
Histidine	10.29	11.32	11.25	13.2	13.65
Serin	51.46	47.89	32.51	48.84	47.11
Glutamine	0.0	0.0	0.0	0.0	0.0
Glysine	83.55	93.25	93.36	92.4	95.24
Threonin	59.54	58.8	69.5	64.47	68.95
Arginin	92.73	102.14	105.45	103.67	103.73
Taurine	10.29	13.84	10.63	13.91	11.83
Alanine	102.02	110.22	112.33	110.98	105.85
Methionine	15.24	16.06	16.26	17.26	20.02
Valine	27.04	41.02	37.41	26.6	36.3
phenylalanine	14.63	10.41	10.84	14.82	19.41
Isoleusine	11.0	16.87	12.5	16.04	12.13
Leusine	20.79	22.02	24.07	21.83	21.43
Lysine	31.38	37.99	29.07	24.37	38.32
Hydroxyproline+ proline	295.76	257.12	262.06	265.0	238.8

ChClGl: Choline chloride- glycerol, Ch Cl Ur: Choline Chloride urea, Ch Cl AA: Choline Chloride Acetic acid, Ch Cl AA US 5: Choline Chloride Acetic acid- 5 min ultrasound, Ch Cl AA US10: Choline Chloride Acetic acid- 5 min ultrasound, Ch Cl AA US15: Choline Chloride Acetic acid- 15 min ultrasound, Ch Cl AA US20: Choline Chloride Acetic acid- 20 min ultrasound.

Values are expressed as number of residues per 1000 total amino acid residues.

### 3.9 Water holding capacity

**Fig. 6:**
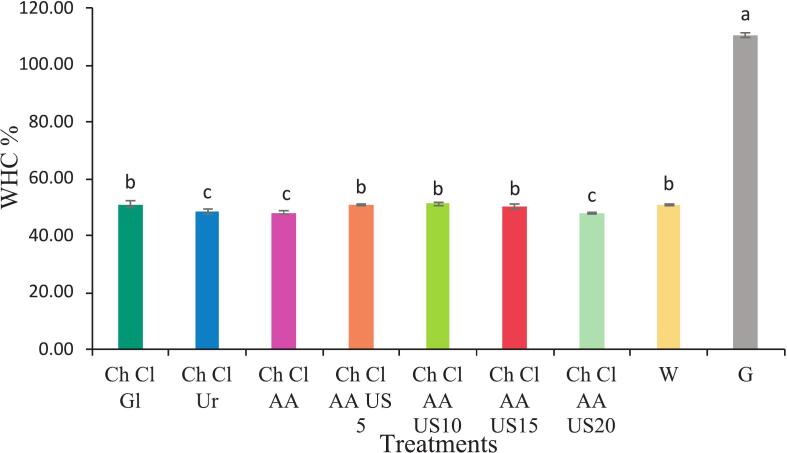
Diagram of water holding capacity of collagen extracted from beluga skin (Ch Cl Gl: Choline chloride- glycerol, Ch Cl Ur: Choline Chloride urea, Ch Cl AA: Choline Chloride Acetic acid, Ch Cl AA US 5: Choline Chloride Acetic acid- 5 min ultrasound, Ch Cl AA US10: Choline Chloride Acetic acid- 5 min ultrasound, Ch Cl AA US15: Choline Chloride Acetic acid- 15 min ultrasound, Ch Cl AA US20: Choline Chloride Acetic acid- 20 min ultrasound, W: water, Gl: Glycerol).

Water holding capacity (WHC) refers to the ability of proteins to retain water within their three-dimensional structure. Multiple factors influence water-holding capacity (WHC), which tends to be at its lowest near the isoelectric point (pI), where the protein carries no net charge, causing protein-protein interactions to dominate. Environmental factors such as protein concentration and ionic strength also impact WHC (Liu et al., 2023). The water-holding capacity of collagen extracted with acetic acid was lower than that of Choline Chloride glycerol, which can be attributed to the low pH (Fig. 6). Generally, WHC is defined as the amount of hydrophilic amino acids present. A lower pH can alter interactions among protein side chains, exposing hydrophobic regions that bind to more surfactant molecules, thereby enhancing the hydrophobic properties of collagen (Shaik et al., 2021). The highest WHC was observed in treatment of 5 min ultrasound. Applying Ultrasound can increase WHC by enhancing the surface hydrophilicity and charged groups of collagens. These results are consistent with those reported by Shaik et al. (2021) (Shaik et al., 2021). Conversely, applying (20 min of ultrasound) exhibited the lowest water holding capacity. Given that there is a direct relationship between WHC and hydrophilic amino acid residues, variations in water-holding capacities may be linked to different amino acid profiles in collagen treatments. This observation aligns with findings from Heidari and Rezaei (2022) (Heidari & Rezaei, 2022).

### 3.10 Gel strength

Gel strength is a critical parameter in evaluating collagen functionality, particularly in applications such as gelatin-based foods, pharmaceutical carriers, and biomedical materials. According to Huang et al. (2016), gel strength values are typically classified into three categories: low (<150 g), medium (150–220 g), and high (220–300 g). Differences in gel strength are influenced by factors such as fish species, collagen source (skin, bone, or scales), amino acid composition, especially imino acids, and the length and integrity of the protein chains (Cao et al., 2020). In our study, collagen samples subjected to 10 min of ultrasound treatment exhibited the highest gel strength (185.00 ± 2.45 g), placing them within the medium-strength category (Table 6). This increase can be attributed to enhanced dispersion of collagen aggregates and stimulation of fibrillogenesis under moderate sonication. During this phase, the collagen triple-helix structure is largely preserved, and sufficient hydrogen bonding enables the formation of a more regular and interconnected fiber network. These structural features contribute to improved gel-forming capacity. However, further increasing the ultrasound time to 20 min led to a significant decline in gel strength. This reduction is likely due to excessive cavitation and mechanical stress, which disrupt collagen's higher-order structure, reduce hydrogen bonding, and fragment the polypeptide chains—hindering their ability to form cohesive networks. This observation is in agreement with previous studies (Liu et al., 2023; Wang et al., 2021), which report that intense or prolonged ultrasound can cause over-denaturation or overstretching of collagen molecules, resulting in weaker gels due to impaired cross-linking and disrupted water-binding interactions. Notably, Liu et al. (2023) suggested that ultrasound-induced molecular unfolding during fibrillogenesis increases hydrophobic interactions while reducing collagen–water binding, ultimately weakening gel strength. Our results are consistent with this dual-effect mechanism: moderate ultrasound enhances structural organization and gel strength, while excessive exposure compromises it. Overall, the observed changes in gel strength across treatments underscore the importance of optimizing ultrasound parameters to balance molecular integrity and functional performance in collagen-based systems.

**Table 6:** The strength of collagen gel extracted from beluga skin.

Treatment	Ch Cl- Gl	Ch Cl- Ur	Ch Cl- AA	Ch Cl- AA 5 min US	Ch Cl- AA 10 min US	Ch Cl- AA 15 min US	Ch Cl- AA 20 min US
Gel strength(g)	140.67 ± 0.94 ^d^	152.33 ± 2.05 ^c^	155.33 ± 0.94 ^c^	159.67 ± 1.25 ^b^	185.00 ± 2.45 ^a^	157.67 ± 1.25 ^bc^	156.67 ± 1.25 ^bc^

ChClGl: Choline chloride- glycerol, Ch Cl Ur: Choline Chloride urea, Ch Cl AA: Choline Chloride Acetic acid, Ch Cl AA US 5: Choline Chloride Acetic acid- 5 min ultrasound, Ch Cl AA US10: Choline Chloride Acetic acid- 5 min ultrasound, Ch Cl AA US15: Choline Chloride Acetic acid- 15 min ultrasound, Ch Cl AA US20: Choline Chloride Acetic acid- 20 min ultrasound.

The results were expressed as mean value ± SD (n = 3). Different letters within the samples mean statistical difference (p < 0.05).

## 4 Conclusion

The successful isolation of type I collagen from beluga sturgeon skin using a choline chloride-acetic acid-based eutectic solvent combined with ultrasound assistance demonstrates an efficient, eco-friendly, and economically viable extraction method. Ultrasonic treatment significantly enhanced collagen yield while preserving its structural integrity, as confirmed by SDS-PAGE and FTIR analyses. Although prolonged ultrasound exposure slightly reduced thermal stability by decreasing imino acid content, it improved functional properties such as water retention and gelation capacity, making the extracted collagen suitable for high-value applications. This study not only introduces beluga sturgeon skin as a sustainable collagen source but also presents a green and scalable extraction technique with potential applications in the food, cosmetic, biomedical, and pharmaceutical industries. The proposed method offers a promising alternative to conventional collagen extraction, aligning with modern demands for sustainable and high-performance biomaterials.

## CRediT authorship contribution statement

**Elahe Taghitabar Malekshah:** Writing – original draft, Investigation. **Masoud Rezaei:** Supervision, Project administration, Funding acquisition, Conceptualization. **Shahab Naghdi:** Writing – review & editing, Formal analysis. **Reza Tahergorabi:** Writing – review & editing.

## Declaration of competing interest

The authors declare that they have no known competing financial interests or personal relationships that could have appeared to influence the work reported in this paper.

## Data Availability

Data will be made available on request.
